# Fracture and mortality outcomes by osteoporosis treatment route in patients with type 2 diabetes and obesity: a propensity-matched registry study

**DOI:** 10.3389/fendo.2026.1688669

**Published:** 2026-02-03

**Authors:** Vanessa Rouach, Ohad Regev, Hilary Gortler, Yona Greenman, Gabriel Chodick, Inbal Goldshtein

**Affiliations:** 1 Institute of Endocrinology, Diabetes, Hypertension and Metabolism, Sourasky Medical Center, Tel Aviv, Israel; 2 Epidemiology Department, School of Public Health, Tel Aviv University, Tel Aviv, Israel; 3 Joyce and Irving Goldman Medical School, Faculty of Health Sciences, Ben-Gurion University of the Negev, Beer Sheva, Israel; 4 Department of Public Health, Faculty of Health Sciences, Ben-Gurion University of the Negev, Beer Sheva, Israel; 5 Gray Faculty of Medical and Health Sciences, Tel Aviv University, Tel Aviv, Israel; 6 Maccabitech Institute for Research and Innovation, Maccabi Healthcare Services, Tel Aviv, Israel; 7 Dina Recanati School of Medicine, Reichman University, Herzliya, Israel

**Keywords:** anti-resorptive treatments, fracture risk, mortality, obesity, osteoporosis, type 2 diabetes mellitus

## Abstract

**Purpose:**

To evaluate the real-world effectiveness of antiresorptive osteoporosis pharmacologic treatments on fracture incidence and survival in patients with type 2 diabetes mellitus (T2DM), with subgroup analysis by treatment route.

**Methods:**

We conducted a retrospective cohort study using a national registry of patients with T2DM and osteoporosis. Patients receiving antiresorptive treatments (oral or intravenous/subcutaneous [IV/SC]) were compared to untreated individuals using propensity score matching. The primary outcomes were major osteoporotic fracture (MOF) and all-cause mortality. Cox proportional hazards models were used to assess associations, including subgroup analyses by BMI and HbA1c.

**Results:**

Among 8,788 matched patients, treatment was associated with significantly lower mortality (adjusted HR 0.86; 95% CI: 0.78–0.94) but not with reduced fracture incidence (HR 1.11; 95% CI: 0.96–1.29). In the treated subgroup (N = 2,960), IV/SC therapy was associated with reduced fracture risk (HR 0.58; 95% CI: 0.35–0.94) but increased mortality (HR 1.63; 95% CI: 1.35–1.96) compared to oral treatment.

**Conclusion:**

In this real-world cohort of patients with type 2 diabetes and obesity, osteoporosis treatment was associated with improved survival but not reduced fracture risk. Injectable therapies offered greater fracture protection but were linked to higher mortality, likely due to confounding by indication. Poor adherence may limit the effectiveness of oral treatments in routine care. These findings underscore the need for individualized osteoporosis management strategies in high-risk diabetic populations and raise important considerations regarding the optimal route of administration in real-world settings.

## 1 Introduction

Type 2 diabetes mellitus (T2DM) is increasingly recognized as a condition associated with elevated fracture risk, despite patients often presenting with normal or even high bone mineral density (BMD) values (1–3). This paradox is attributed to compromised bone quality, impaired bone turnover, and the presence of microvascular complications, all of which contribute to skeletal fragility in diabetic individuals (2, 3). Moreover, obesity—frequently coexisting with T2DM—complicates the relationship between body composition, bone health, and fracture risk. While a higher body mass index (BMI) has historically been viewed as protective against osteoporosis, emerging data suggest that obesity may increase the risk of certain fracture types and negatively impact bone architecture (4).

Pharmacologic treatment for osteoporosis, including oral bisphosphonates, intravenous bisphosphonates, and monoclonal antibodies such as denosumab, has demonstrated efficacy in reducing fracture risk in the general population (5–9). However, patients with diabetes and/or obesity are often underrepresented in clinical trials, and limited evidence exists regarding the real-world effectiveness of osteoporosis therapies in these high-risk subgroups (10–13). Furthermore, uncertainty remains regarding the relative benefits of different treatment modalities—particularly injectable versus oral agents—and their impact on both skeletal and survival outcomes in patients with metabolic comorbidities (14–16).

To address this gap, we conducted a large, registry-based, propensity score–matched study to evaluate the association between osteoporosis treatment and fracture incidence as well as overall survival in patients with T2DM. We further explored outcomes according to treatment route (intravenous/subcutaneous vs. oral) and examined whether specific subgroups, such as those with obesity or impaired glycemic control, experienced differential effects.

## 2 Subjects and methods

The study utilized longitudinal data from Maccabi Healthcare Services (MHS). MHS is the second largest healthcare provider and insurer in Israel, covering over two million members, which accounts for approximately 25% of the population with a countrywide distribution. The insured population is nationally representative because according to the 1995 national health insurance law, health medical organizations (HMOs) may not deny coverage to applicants on any grounds, including age or state of health. MHS’s central database contains patient demographics, diagnoses, medical procedures, hospitalizations, and full capture of all prescription medication dispensations and laboratory tests since 1999. MHS has developed several computerized registries of major chronic diseases, such as oncologic diseases, diabetes, and osteoporosis that are continuously updated (17, 18). The study follow-up period extended from January 1998 to December 2021.

The current study obtained data from the MHS diabetes and osteoporosis registries. Registry assembly has been previously described elsewhere, and a comprehensive approach was used to cross-validate them and ensure high specificity (19).

Briefly, the osteoporosis registry identifies patients by diagnoses, by at least two dispensations of medications for osteoporosis, by bone mineral density (BMD) in the osteoporotic range (*T*-score ≤−2.5), or by a major osteoporotic fracture (MOF) which occurred at a typical age (50+ years for females and 60+ years for males). Registry entry date is the earliest of all the above criteria.

Major osteoporotic fractures (MOF) were identified using ICD-9/ICD-10 diagnostic codes recorded in the Maccabi Healthcare Services electronic medical records, clinician diagnoses, and records of fracture-related procedures. MOF sites included the hip, spine, distal radius, and proximal humerus fractures, in accordance with fracture risk assessment definitions (20). Only low-trauma fractures, excluding those related to high-energy trauma or pathological fractures, were included. Our data source does not include access to radiographic images or structured radiology reports; therefore, fractures could only be identified through ICD diagnostic codes and clinical documentation within the electronic medical record.

The diagnosis codes were verified using internal registry validation processes previously described in MHS epidemiological research (19).

The diabetes registry identifies patients according to HbA1c values and glucose test results, DM therapy dispensations, and a diagnosis of DM from relevant physicians, with an overall specificity of 99.99%. Considering the different pathogeneses of type 1 and type 2 diabetes, the current study focused on type 2 diabetes compared to no diabetes.

### 2.1 Participants

The study population was identified through cross-linkage of the Maccabi Healthcare Services (MHS) diabetes registry and osteoporosis registry. Eligible participants were men and women aged over 50 years who had entered the diabetes registry prior to their osteoporosis diagnosis. The index date was defined as osteoporosis registry entry date. As treatment duration was not consistently recorded and could not be reliably assessed, the analysis was based on treatment initiation rather than cumulative exposure. Patients receiving other osteoporosis treatments or with a history of cancer, multiple myeloma, or Paget’s disease of bone were excluded. As previously described, inclusion in the osteoporosis registry was based on documentation of bone mineral density (BMD) in the osteoporotic range, a major osteoporotic fracture (MOF), or initiation of anti-osteoporotic therapy. None of the included participants had a history of prior fractures or previous anti-osteoporotic treatment.

### 2.2 Outcomes

The primary outcome was MOF (hip, spine, distal radius, and proximal humerus fractures).

The secondary outcome was death.

### 2.3 Additional covariates

We extracted data on demographic and clinical variables, including age, body mass index (BMI), hemoglobin A1c levels, diabetes duration, presence of microvascular and macrovascular complications, insulin use, history of hypoglycemic events, falls, estimated glomerular filtration rate (eGFR), Charlson Comorbidity Index (CCI), and the purchase of statins, and glucose-lowering agents. Additional variables included bone mineral density (BMD) T-scores, smoking history, alcohol consumption, diagnoses of rheumatoid arthritis, and chronic glucocorticoid use. Most variables were captured at the time of entry into both the diabetes and osteoporosis registries; for the purposes of this analysis, we used the values recorded at the time of osteoporosis registry entry.

### 2.4 Statistical analysis

Descriptive statistics were employed to characterize the study population. Categorical variables were presented as frequencies and percentages, while numerical variables were presented as mean ± standard deviation (SD) for normally distributed data or median with interquartile range (IQR) for ordinal or non-normally distributed data. Normality of continuous variables was assessed using histogram plots, Shapiro–Wilk test, and Kolmogorov–Smirnov test.

Univariate analysis was conducted to compare clinical and sociodemographic characteristics between patients receiving treatment to those who did not as well as between treatment types. For continuous variables with normal distribution, independent sample t-test was used. For continuous variables with non-normal distribution and ordinal variables, Mann–Whitney U test was employed. For categorical variables, chi-square test or Fisher’s exact test was used as appropriate.

Propensity score matching was employed to address the baseline differences in patients’ characteristics, including age, sex, BMI, diabetes duration, Charlson comorbidity index, T-score, insulin treatment, hypoglycemic events, microvascular complications, and history of falls. The *MatchIt* package in R was utilized to calculate the propensity score for each patient and conduct propensity score matching between the two patient groups using nearest-neighbor matching with a caliper of 0.1. Matching ratios were 1:1 for treatment comparison (treated vs untreated) and 1:4 for treatment type comparison (IV/SC vs PO). Post-matching covariate balance was evaluated using standardized mean differences (SMD) displayed in Love plots (**Supplementary Figures S1**, **S2**), with successful matching defined as achieving SMD values <0.1 for all covariates, indicating adequate balance between groups.

To evaluate the time-dependent associations between osteoporosis treatment and clinical outcomes, we used time-dependent Cox models and Simon–Makuch curves. These analytical approaches allow for dynamic classification of treatment status, capturing the real-world scenario in which patients initiated therapy at different time points following osteoporosis registry entry, while others remained untreated throughout follow-up. This methodology properly addresses potential immortal time bias by ensuring that patients contribute follow-up time to both untreated and treated periods as their exposure status changes. In a secondary analysis restricted to patients who received treatment, we evaluated associations between treatment type (IV/SC vs PO) and clinical outcomes using log-rank tests, Kaplan–Meier curves, and standard Cox models. Since all patients in this subset received treatment, time-dependent methodology was not required. For both analyses, univariate and multivariable Cox models were used to estimate adjusted hazard ratios (HR) and 95% confidence intervals (CIs) for each outcome while adjusting for potential confounders. The proportional hazards assumption was assessed graphically and using Schoenfeld residuals. Analyses were conducted for the entire cohort and stratified by patients’ age, BMI, HbA1c, and eGFR. All survival analyses were performed using the *Survival* and *Survminer* packages in R.

All statistical analyses were performed using SPSS Statistics version 25 and R software. A two-sided test significance level of p<0.05 was used throughout the entire study.

### 2.5 Ethical approval

The study protocol was approved by the HMO Institutional Review Board. All procedures were in accordance with the Declaration of Helsinki, and patient data were anonymized prior to analysis; no individual consent was required.

## 3 Results

### 3.1 Baseline characteristics

A total of 11,716 patients with diabetes and osteoporosis were included in the study cohort, of whom 6,490 (55.4%) received pharmacologic osteoporosis treatment and 5,226 (44.6%) did not. Treated patients were more likely to be female (71.0% vs. 63.1%, *p* < 0.001) and had a slightly lower mean BMI (29.6 ± 5.2 vs. 30.6 ± 5.4 kg/m², *p* < 0.001). No significant differences were observed between groups in age at diabetes diagnosis (63.4 ± 7.6 years vs. 63.3 ± 7.7, *p* = 0.265) or in HbA1c levels (6.7 ± 0.8% in both groups, *p* = 0.002), although treated patients had a shorter median diabetes duration (5.1 vs. 8.0 years, *p* < 0.001). Osteoporosis diagnosis occurred at a younger age in treated individuals (69.9 ± 7.7 vs. 72.0 ± 7.7 years, *p* < 0.001), and their BMD T-scores were significantly lower across all measured sites, including the left hip (median −2.0 vs. −1.6, *p* < 0.001), total hip (median -1.4 vs. -1.0, *p* < 0.001), and lumbar spine (median -1.5 vs. -0.8, *p* < 0.001). Treated patients had higher rates of insulin use (26.6% vs. 21.6%, *p* < 0.001), microvascular complications (69.3% vs. 63.6%, *p* < 0.001), and more hypoglycemic events (8.0% vs. 7.0%, *p* = 0.046). There were no statistically significant differences in history of falls, hip fractures at osteoporosis registry entry, statin use, or GFR levels between groups (**Table 1**).

**Table 1:** Sociodemographic and clinical characteristics of study cohort stratified by osteoporosis treatment.

Variable		Overall (N = 11,716)	Tx (N = 6,490)	No Tx (N = 5,226)	P-value
Sex ^1^	Female	7,911(67.5)	4,611(71.0)	3,300(63.1)	**<0.001 ^a^**
	Male	3,805(32.5)	1,879(29.0)	1,926(36.9)	
Smoking ^1,4^		427(9.0)	211(10.0)	216(8.2)	**0.026 ^a^**
BMI ^2^		30.0 ± 5.3	29.6 ± 5.2	30.6 ± 5.4	**<0.001 ^b^**
Alcohol use ^1^		60(0.5)	36(0.6)	24(0.5)	0.472 ^a^
Age at DM diagnosis ^2^		63.4 ± 7.6	63.4 ± 7.6	63.3 ± 7.7	0.265 ^b^
Diabetes duration ^3^		6.3(2.6, 11.4)	5.1(2.0, 9.7)	8.0(3.6, 13.1)	**<0.001 ^c^**
HA1C ^2,4^	At DM diagnosis	6.7 ± 0.8	6.7 ± 0.8	6.7 ± 0.8	**0.002 ^c^**
	At OP diagnosis	6.8 ± 1.1	6.8 ± 1.0	6.8 ± 1.1	0.186 ^c^
Insulin treatment ^1^		2,855(24.4)	1,726(26.6)	1,129(21.6)	**<0.001 ^a^**
Statins treatment ^1^		4,552(38.9)	2,542(39.2)	2,010(38.5)	0.436 ^a^
Age at OP diagnosis ^2^		70.8 ± 7.8	69.9 ± 7.7	72.0 ± 7.7	**<0.001 ^b^**
CCI ^3^	At DM diagnosis	1(0, 2)	1(0, 2)	1(0, 2)	0.456 ^c^
	At OP diagnosis	3(1, 5)	3(1, 5)	3(1, 5)	0.219 ^c^
BMD T-score ^3,4^	Femoral neck	−1.8(−2.4, −1.2)	−2.0(−2.5, −1.4)	−1.6(−2.2, −0.9)	**<0.001 ^c^**
	Total hip	−1.3(−1.9, −0.6)	−1.4(−2.0, −0.8)	−1.0(−1.7, −0.3)	**<0.001 ^c^**
	Lumbar spine	−1.1(−2.1, 0.0)	−1.5(−2.3, −0.4)	−0.8(−1.8, 0.4)	**<0.001 ^c^**
Hypoglycemic events ^1^		884(7.5)	518(8.0)	366(7.0)	**0.046 ^a^**
Microvascular complications ^1^		7,823(66.8)	4,498(69.3)	3,325(63.6)	**<0.001 ^a^**
Falls ^1^		2,399(20.5)	1,364(21.0)	1,035(19.8)	0.106 ^a^
Hip fracture at OP diagnosis ^1^		296(2.5)	128(2.4)	168(2.6)	0.633 ^a^
eGFR ^3,4^		73(54, 88)	71(54, 87)	75(55, 88)	**0.081 ^c^**

Boldface type indicates p<0.05.

^1^Number (%); ^2^mean ± standard deviation; ^3^median (interquartile range).

^4^Number of patients with missing data: smoking - 6,975(59.4%); HA1C at index 2—9,654(82.4%); left hip total T score—1,297(11.1%), L_2_L_4_ T score—5,927(50.6%); GFR—9,121(77.9%).

^a^Chi-square; ^b^independent sample T-test; ^c^Mann–Whitney.

BMI, body mass index; BMD, bone mineral density; CCI, Charlson Comorbidity Index; DM, diabetes mellitus; eGFR, estimated glomerular filtration rate; HbA1c, glycated hemoglobin; OP, osteoporosis.

**Figure 1:**
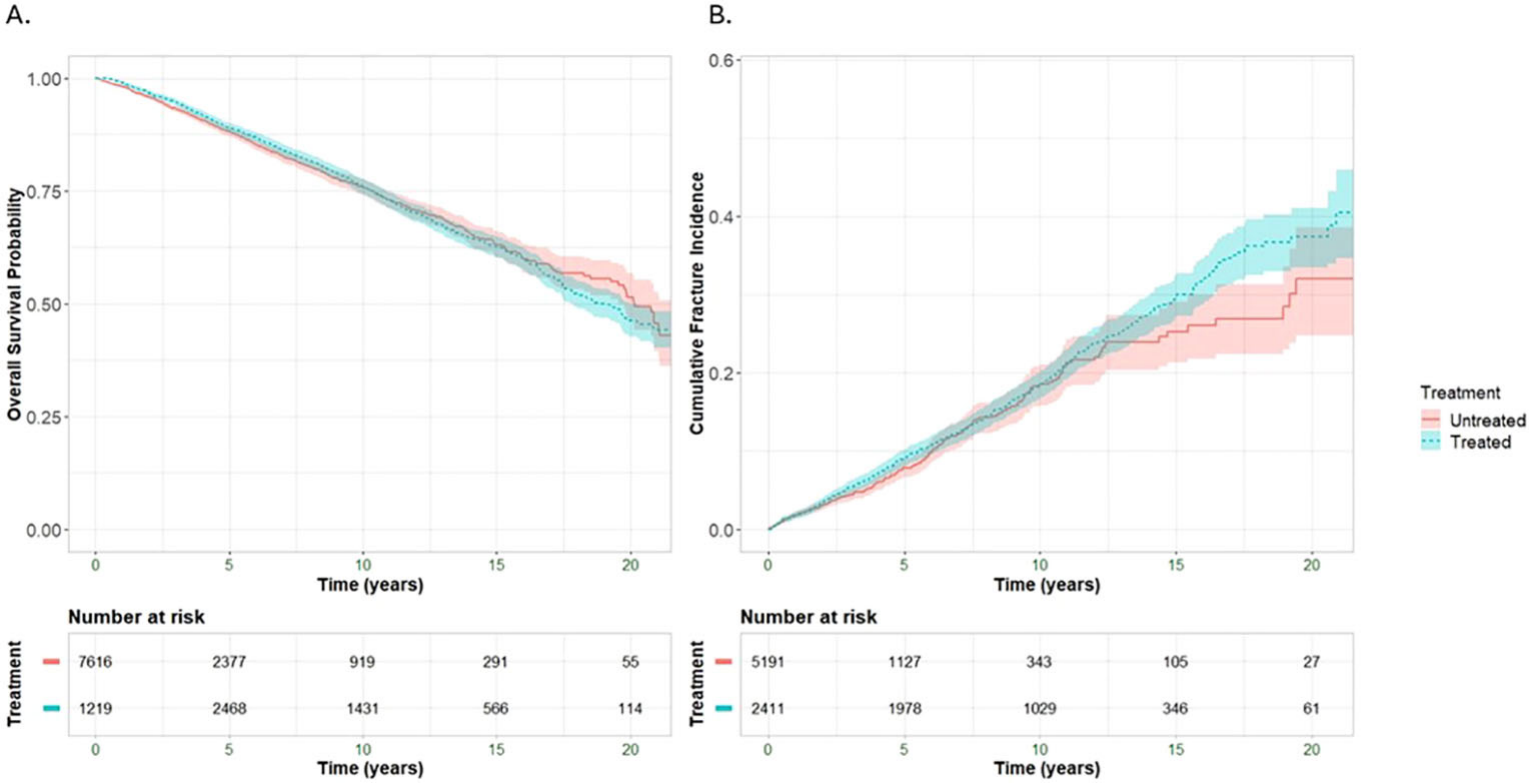
Simon–Makuch curves showing **(A)** overall survival and **(B)** cumulative fracture incidence following osteoporosis diagnosis. Blue lines represent patients during treated periods; red lines represent patients during untreated periods. Shaded areas indicate 95% confidence intervals. This time-dependent analysis accounts for treatment initiation timing, with patients contributing to both groups as their treatment status changes over follow-up.

To evaluate the time-dependent associations between osteoporosis treatment and clinical outcomes, we constructed Simon–Makuch survival curves. These plots allow for dynamic classification of treatment status, capturing the real-world scenario in which some patients initiated therapy following osteoporosis registry entry, while others remained untreated. Survival probability remained comparable during the initial years but began to diverge around year 5, with patients in treated periods demonstrating improved overall survival over time. This association persisted through long-term follow-up, suggesting a potential survival benefit linked to treatment initiation (**Figure 1A**). Although fracture rates increased in both groups over time, the incidence was modestly higher during treated periods (**Figure 1B**).

Following 1:1 propensity score matching, the final cohort included 8,788 patients, with 4,394 individuals in both the treated and untreated groups. Baseline characteristics were well-balanced between groups for most clinical and sociodemographic variables. There were no significant differences in sex distribution (female: 65.0% treated vs. 64.9% untreated, *p* = 0.947), mean BMI (30.2 ± 5.4 vs. 30.2 ± 5.2 kg/m², *p* = 0.646), age at diabetes diagnosis (63.5 ± 7.6 vs. 63.6 ± 7.8 years, *p* = 0.676), HbA1c levels (6.7 ± 0.7 vs. 6.7 ± 0.8%, *p* = 0.463), or Charlson Comorbidity Index (median 1 [IQR 0–2] in both groups). Compared to untreated patients, treated individuals had modestly lower bone mineral density across all measured sites: femoral neck T-score (median −1.9 vs. −1.7, *p* < 0.001), total hip (median −1.3 vs. −1.2, *p* < 0.001), and lumbar spine (median −1.3 vs. −1.0, *p* < 0.001). A small but statistically significant difference was observed in estimated glomerular filtration rate (median 72 vs. 76 mL/min/1.73 m², *p* = 0.017), although both groups were within the same clinical range. Rates of insulin use (22.6% vs. 22.5%, *p* = 0.980), microvascular complications (64.8% vs. 64.5%, *p* = 0.789), hypoglycemic events, falls, and hip fractures at OP diagnosis were comparable between groups (**Table 2**).

**Table 2:** Sociodemographic and clinical characteristics of study cohort stratified by osteoporosis treatment—propensity score matched cohort.

Variable		Overall (N = 8,788)	Tx (N = 4,394)	No Tx (N = 4,394)	P-value
Sex ^1^	Female	5,705(64.9)	2,854(64.9)	2,854(65.0)	0.947 ^a^
	Male	3,083(35.1)	1,543(35.1)	1,540(35.0)	
Smoking ^1,4^		320(9.0)	134(7.5)	186(10.4)	**0.003 ^a^**
BMI ^2^		30.2 ± 5.3	30.2 ± 5.4	30.2 ± 5.2	0.646 ^b^
Alcohol use ^1^		51(0.6)	31(0.7)	20(0.5)	0.122 ^a^
Age at DM diagnosis ^2^		63.6 ± 7.7	63.5 ± 7.6	63.6 ± 7.8	0.676 ^b^
Diabetes duration ^3^		6.7(3.0, 11.5)	6.6(3.0, 11.5)	6.8(3.0, 11.5)	0.210 ^c^
HA1C ^2,4^	At DM diagnosis	6.7 ± 0.8	6.7 ± 0.7	6.7 ± 0.8	0.463 ^c^
	At OP diagnosis	6.8 ± 1.1	6.8 ± 1.0	6.8 ± 1.1	0.422 ^c^
Insulin treatment ^1^		1981(22.5)	991(22.6)	990(22.5)	0.980 ^a^
Statins treatment ^1^		3,414(38.8)	1,710(38.9)	1,704(38.8)	0.896 ^a^
Age at OP diagnosis ^2^		71.2 ± 7.6	71.1 ± 7.6	71.3 ± 7.6	0.223 ^b^
CCI ^3^	At DM diagnosis	1(0, 2)	1(0, 2)	1(0, 2)	0.157 ^c^
	At OP diagnosis	3(1, 5)	3(1, 5)	3(1, 5)	0.984 ^c^
BMD T-score ^3,4^	Femoral neck	−1.8(−2.4, −1.2)	−1.9(−2.5, −1.3)	−1.7(−2.3, −1.1)	**<0.001 ^c^**
	Total hip	−1.2(−1.9, −0.6)	−1.3(−1.9, −0.7)	−1.2(−1.8, −0.5)	**<0.001 ^c^**
	Lumbar spine	−1.1(−2.0, 0.0)	−1.3(−2.2, −0.2)	−1.0(−1.9, 0.1)	**<0.001 ^c^**
Hypoglycemic events ^1^		623(7.1)	315(7.2)	308(7.0)	0.771 ^a^
Microvascular complications ^1^		5,682(64.7)	2,847(64.8)	2,835(64.5)	0.789 ^a^
Falls ^1^		1,784(20.3)	901(20.5)	883(20.1)	0.633 ^a^
Hip fractures at OP diagnosis ^1^		217(2.5)	104(2.4)	113(2.6)	0.536 ^a^
eGFR ^3,4^		74(55, 88)	72(54, 87)	76(56, 88)	0.017 ^c^

Boldface type indicates p<0.05.

^1^Number (%); ^2^mean ± standard deviation; ^3^median (interquartile range).

^4^Number of patients with missing data: smoking—5,214(59.3%); HA1C at osteoporosis diagnosis—7,189(81.8%); left hip total T score—913(10.4%), L_2_L_4_ T score—4,192(47.7%); GFR—6,737(76.7%).

^a^Chi-square; ^b^Independent sample T-test; ^c^Mann–Whitney.

BMI, body mass index; BMD, bone mineral density; CCI, Charlson Comorbidity Index; DM, diabetes mellitus; eGFR, estimated glomerular filtration rate; HbA1c, glycated hemoglobin; OP, osteoporosis.

### 3.2 Association between osteoporosis treatment and patient outcomes

In the propensity score–matched cohort, osteoporosis treatment was significantly associated with improved survival but not with a reduction in fracture risk.

In multivariable time-dependent Cox regression models, treatment was associated with a 14% lower risk of death (adjusted HR = 0.86, 95% CI: 0.78–0.94, *p* < 0.001), after adjusting for left hip T-score, sex, and age at diabetes and osteoporosis diagnoses. However, no significant association was observed between treatment and fracture incidence (HR = 1.11, 95% CI: 0.96–1.29, *p* = 0.174).

Stratified analyses showed that the survival benefit persisted across subgroups:

- Among patients aged >75 years, treatment was associated with a 22% lower mortality risk (HR = 0.78, 95% CI: 0.68–0.89, *p* < 0.001).
- In all BMI categories, treatment remained protective (HR = 0.79, 0.83, and 0.80, respectively, p<0.001, 0.040, and 0.049 respectively).
- Among those with HbA1c <8%, treatment was associated with improved survival (HR = 0.76, 95% CI: 0.60–0.97, *p* = 0.026), whereas no benefit was seen in patients with poor glycemic control (HbA1c ≥8%).
- Notably, survival benefit was strongest in patients with impaired renal function: HR = 0.53 (95% CI: 0.33–0.83, *p* = 0.005) for eGFR <40 mL/min/1.73m² and HR = 0.42 (95% CI: 0.20–0.86, *p* = 0.017) for eGFR <30.

No significant associations were found between treatment and fracture incidence across any subgroup (**Table 3**).

**Table 3:** Association between treatment to patient survival and fracture event—propensity score matched cohort.

Variable	Group	Survival			Fracture event		
		Adjusted HR ^a^	95% CI	Pv	Adjusted HR ^a^	95% CI	Pv
**Overall**		0.86	0.78-0.94	**<0.001**	1.11	0.96-1.29	0.174
**Age ^1^**	Below 75	0.94	0.83-1.07	0.372	1.10	0.92-1.33	0.296
	Above 75	0.78	0.68-0.89	**<0.001**	1.12	0.86-1.46	0.390
**BMI ^2^**	Below 30	0.79	0.69-0.90	**<0.001**	1.08	0.88-1.33	0.472
	30-35	0.83	0.70-0.99	**0.040**	1.17	0.90-1.53	0.238
	Above 35	0.80	0.63-0.99	**0.049**	1.03	0.71-1.49	0.877
**HbA1C ^3^**	Below 8	0.76	0.60-0.97	**0.026**	0.95	0.64-1.40	.780
	Above 8	0.68	0.33-1.39	0.288	0.99	0.30-3.35	0.996
**eGFR ^4^**	Above 40	0.88	0.68-1.13	0.321	1.18	0.77-1.81	0.444
	Below 40	0.53	0.33-0.83	**0.005**	0.62	0.26-1.47	0.277
	Below 30	0.42	0.20-0.86	**0.017**	1.65	0.47-5.80	0.436

Boldface type indicates p<0.05.

HR, hazard ratio; CI, confidence interval; Pv = P-value.

^a^Multivariable time-dependent Cox regression, adjusted to left hip T score, patient sex, and age at DM and osteoporosis diagnosis.

Number of patients in each group: ^1^Below 75 = 6,108, above 75 = 2,680; ^2^below 30 = 4,744, above 30 = 4,044, above 35 = 1,481; ^3^below 8 = 1,449, above 8 = 150; ^4^below 30 = 83, below 40 = 177, above 40 = 1,874.

BMI, body mass index; CCI, Charlson Comorbidity Index; CI, confidence interval; eGFR, estimated glomerular filtration rate; HbA1c, glycated hemoglobin; HR, hazard ratio.

### 3.3 Comparison by treatment route

Among treated patients (N = 6,490), 606 (9.3%) received intravenous or subcutaneous (IV/SC) osteoporosis therapy and 5,884 (90.7%) received oral (PO) treatment.

Patients in the IV/SC group were more likely to be male (39.1% vs. 27.9%, *p* < 0.001) and had a longer median diabetes duration (7.2 vs. 5.0 years, *p* < 0.001) and a lower mean HbA1c at diabetes diagnosis (6.6% vs. 6.8%, *p* < 0.001). They were also older at the time of osteoporosis diagnosis (72.8 ± 7.7 vs. 69.6 ± 7.7 years, *p* < 0.001), suggesting more advanced disease at treatment initiation. BMD measurements indicated slightly lower total hip T-scores among IV/SC users (median −1.5 vs. −1.4, *p* = 0.003), while lumbar spine T-scores were notably higher in this group (median −1.1 vs. −1.5, *p* < 0.001). No significant differences were observed in femoral neck T-scores (both groups median −2.0, *p* = 0.071). Rates of insulin use, statin use, and microvascular complications were similar between groups. However, hip fractures at osteoporosis registry entry were more frequent in the IV/SC group (5.4% vs. 2.3%, *p* < 0.001), and median GFR values were slightly lower (68 vs. 72 mL/min/1.73 m², *p* = 0.033), possibly indicating greater baseline risk (**Table 4**).

**Table 4:** Sociodemographic and clinical characteristics of study cohort stratified by treatment route.

Variable		Overall (N = 6,490)	PO (N = 5,884)	IV/SC (N = 606)	P-value
Sex ^1^	Female	1,879(29.0)	4,242(72.1)	369(60.9)	**<0.001 ^a^**
	Male	4,611(71.0)	1,642(27.9)	237(39.1)	
Smoking ^1,4^		216(8.2)	196(8.2)	20(7.9)	0.865 ^a^
BMI ^2^		29.6 ± 5.2	29.6 ± 5.2	29.5 ± 5.2	0.801 ^b^
Alcohol use ^1^		36(0.6)	34(0.6)	2(0.3)	0.771 ^d^
Age at DM diagnosis ^2^		63.5 ± 7.6	63.4 ± 7.5	64.6 ± 8.0	**<0.001 ^b^**
Diabetes duration ^3^		5.1(2.0, 9.7)	5.0(2.0, 9.4)	7.2(3.3, 12.8)	**<0.001 ^c^**
HA1C ^2,4^	At DM diagnosis	6.7 ± 0.8	6.8 ± 0.8	6.6 ± 0.7	**<0.001 ^c^**
	At OP diagnosis	6.8 ± 1.0	6.8 ± 1.1	6.6 ± 1.1	**0.017 ^c^**
Insulin treatment ^1^		1,726(26.6)	1,554(26.4)	172(28.4)	0.295 ^a^
Statins treatment ^1^		2,542(39.2)	2,299(39.1)	243(40.1)	0.622 ^a^
Age at OP diagnosis ^2^		69.9 ± 7.7	69.6 ± 7.7	72.8 ± 7.7	**<0.001 ^b^**
CCI ^3^	At DM diagnosis	1(0, 2)	1(0, 2)	1(0, 2)	0.444 ^c^
	At OP diagnosis	3(1, 5)	3(1, 5)	3(1, 5)	0.646 ^c^
BMD T-score ^3,4^	Femoral neck	−2.0(−2.5, −1.4)	−2.0(−2.5, −1.4)	−2.0(−2.6, −2.0)	0.071 ^c^
	Total hip	−1.4(−2.0, −0.8)	−1.4(−2.0, −0.8)	−1.5(−2.3, −1.5)	**0.003 ^c^**
	Lumbar spine	−1.5(−2.3, −0.4)	−1.5(−2.3, −0.4)	−1.1(−2.1, 0.0)	**<0.001 ^c^**
Hypoglycemic events ^1^		518(8.0)	467(7.9)	51(8.4)	0.679 ^a^
Microvascular complications ^1^		4,498(69.3)	4,093(69.6)	405(66.8)	0.165 ^a^
Falls ^1^		1,364(21.0)	1,223(20.8)	141(23.3)	0.153 ^a^
Hip fractures at OP diagnosis ^1^		170(2.6)	137(2.3)	33(5.4)	**<0.001 ^a^**
eGFR ^3,4^		71(54, 87)	72(54, 88)	68(47, 86)	**0.033 ^c^**

Boldface type indicates p<0.05.

^1^Number (%); ^2^mean ± standard deviation; ^3^median (interquartile range).

^a^Chi-square; ^b^independent sample T-test; ^c^Mann–Whitney; ^d^Fisher exact test.

BMI, body mass index; BMD, bone mineral density; CCI, Charlson Comorbidity Index; DM, diabetes mellitus; eGFR, estimated glomerular filtration rate; HbA1c, glycated hemoglobin; IV/SC, intravenous or subcutaneous; OP, osteoporosis; PO, oral.

To explore whether route of administration influences clinical outcomes, we compared patients treated with oral (PO) versus intravenous or subcutaneous (IV/SC) antiresorptive therapies. The analysis was restricted to individuals who initiated treatment following osteoporosis diagnosis.

**Figure 2:**
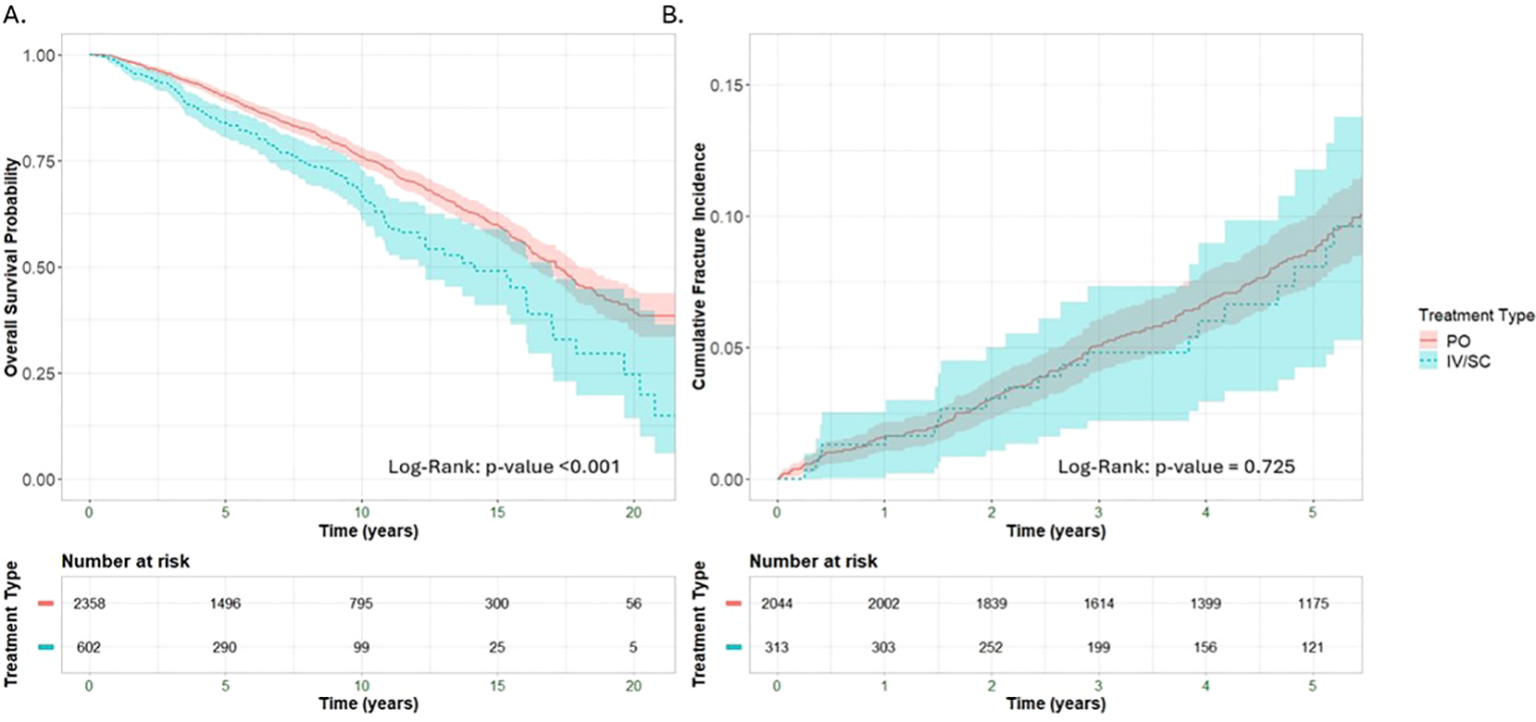
Kaplan–Meier curves showing **(A)** overall survival and **(B)** cumulative 5-year fracture incidence following osteoporosis diagnosis. Blue lines represent patients receiving treatment IV/IM; red lines represent patients receiving treatment PO. Shaded areas indicate 95% confidence intervals. P-value of log-rank test.

Patients treated with injectable therapies (IV/SC) exhibited significantly lower survival compared to those treated with oral agents (PO), with divergence emerging early and a log-rank p-value <0.001 (**Figure 2A**). No statistically significant difference was observed in the cumulative 5-year incidence of major osteoporotic fractures between patients treated with oral and injectable therapies (log-rank p = 0.725), indicating similar fracture protection during the observed follow-up period (**Figure 2B**).

### 3.4 Comparison of treatment route in the propensity score-matched cohort

In the matched cohort of treated patients (N = 2,960), 602 (20.3%) received intravenous/subcutaneous (IV/SC) therapy and 2,358 (79.7%) received oral (PO) osteoporosis treatment. Baseline characteristics were well balanced across treatment groups. There were no significant differences between IV/SC and PO groups in sex, BMI, smoking status, alcohol use, age at diabetes or osteoporosis diagnosis, diabetes duration, or comorbidity burden (CCI). Rates of insulin and statin use, as well as microvascular complications, were also comparable. Patients in the IV/SC group had slightly lower HbA1c at diabetes diagnosis (6.6% vs. 6.7%, *p* < 0.001) and marginally lower HbA1c at the time of osteoporosis diagnosis (6.6% vs. 6.8%, *p* = 0.011). No statistically significant differences were observed in BMD T-scores at the femoral neck, total hip, or lumbar spine. Rates of falls, hypoglycemic events, femoral fractures, and renal function (eGFR) were similar between treatment groups (**Table 5**).

**Table 5:** Sociodemographic and clinical characteristics of study cohort stratified by treatment route—propensity score matched cohort.

Variable		Overall (N = 2,960)	PO (N = 2,358)	IV/SC (N = 602)	P-value
Sex ^1^	Female	1,820(61.5)	1,452(61.6)	368(61.1)	0.840 ^a^
	Male	1140(38.5)	906(38.4)	234(38.9)	
Smoking ^1^		93(7.5)	73(7.3)	20(8.0)	0.934 ^a^
BMI ^2^		29.5 ± 5.2	29.5 ± 5.2	29.5 ± 5.2	0.722 ^b^
Alcohol use ^1^		17(0.6)	15(0.6)	2(0.3)	0.550 ^d^
Age at DM diagnosis ^2^		64.5 ± 7.8	64.5 ± 7.8	64.5 ± 8.0	0.997 ^b^
Diabetes duration ^3^		7.2(3.2, 12.2)	7.3(3.1, 12.1)	7.1(3.2, 12.7)	0.633 ^c^
HA1C ^2^	At DM diagnosis	6.7 ± 0.8	6.7 ± 0.8	6.6 ± 0.7	**<0.001 ^c^**
	At OP diagnosis	6.8 ± 1.0	6.8 ± 1.0	6.6 ± 1.1	**0.011 ^c^**
Insulin treatment^1^		829(28.0)	659(27.9)	170(28.2)	0.887 ^a^
Statins treatment ^1^		1,192(40.3)	952(40.4)	240(39.9)	0.821 ^a^
Age at osteoporosis diagnosis ^2^		72.6 ± 7.4	72.5 ± 7.3	72.7 ± 7.6	0.612 ^b^
CCI ^3^	At DM diagnosis	1(0, 2)	1(0, 2)	1(0, 2)	0.273 ^c^
	At OP diagnosis	3(1, 5)	3(1, 5)	3(1, 5)	0.504 ^c^
BMD T-score ^3^	Femoral neck	−2.1(−2.6, −1.5)	−2.1(−2.6, −1.5)	−2.0(−2.6, −1.4)	0.412 ^c^
	Total hip	−1.5(−2.1, −0.9)	−1.5(−2.1, −0.9)	−1.5(−2.3, −0.9)	0.220 ^c^
	Lumbar spine	−1.3(−2.1, −0.2)	−1.3(−2.2, −0.2)	−1.1(−2.1, 0.0)	0.170 ^c^
Hypoglycemic events ^1^		256(8.6)	206(8.7)	50(8.3)	0.737 ^a^
Microvascular complications ^1^		1,961(66.3)	1,560(66.2)	401(66.6)	0.834 ^a^
Falls ^1^		668(22.6)	529(22.4)	139(23.1)	0.731 ^a^
Hip fractures at OP diagnosis^1^		118(4.0)	88(3.7)	30(5.0)	0.161 ^a^
eGFR ^3^		69(53, 86)	70(54, 86)	68(47, 86)	0.248 ^c^

Boldface type indicates p<0.05.

^1^Number (%); ^2^mean ± standard deviation; ^3^median (interquartile range).

^a^Chi-square; ^b^independent sample T-test; ^c^Mann–Whitney; ^d^Fisher exact test.

BMI, body mass index; BMD, bone mineral density; CCI, Charlson Comorbidity Index; DM, diabetes mellitus; eGFR, estimated glomerular filtration rate; HbA1c, glycated hemoglobin; IV/SC, intravenous or subcutaneous; OP, osteoporosis; PO, oral.

### 3.5 Association between IV/SC treatment and outcomes in the matched cohort

In the propensity score–matched cohort, use of intravenous/subcutaneous (IV/SC) osteoporosis therapy was significantly associated with a lower risk of fracture but also with higher mortality compared to oral (PO) therapy. Over 5 years of follow-up, patients receiving IV/SC treatment had a 42% lower risk of fracture compared to those receiving PO therapy (adjusted HR = 0.58, 95% CI: 0.35–0.94, *p* = 0.028). This association remained significant in patients with BMI >35 (HR = 0.14, 95% CI: 0.03–0.70, *p* = 0.016), but not in other subgroups.

Paradoxically, IV/SC treatment was associated with an increased risk of death compared to PO treatment (adjusted HR = 1.63, 95% CI: 1.35–1.96, *p* < 0.001). This finding was consistent across subgroups, including BMI <30: HR = 1.61 (95% CI: 1.27–2.05, *p* < 0.001), BMI ≥30: HR = 1.62 (95% CI: 1.13–2.32, *p* = 0.008) and HbA1c <8%: HR = 2.02 (95% CI: 1.34–3.03, *p* = 0.001). Among patients with HbA1c ≥8%, the association with mortality did not reach statistical significance (HR = 2.28, 95% CI: 0.71–7.33, *p* = 0.164), possibly due to the small sample size in this subgroup (**Table 6**).

**Table 6:** Association between IV/SC treatment route to patient survival and fracture event—propensity score matched cohort.

Variable	Group	Survival			5-year fracture event		
		Adjusted HR ^a^	95% CI	Pv	Adjusted HR ^b^	95% CI	Pv
**Overall**		1.63	1.35-1.96	**<0.001**	0.58	0.35-0.94	**0.028**
**BMI ^1^**	Below 30	1.61	1.27-2.05	**<0.001**	0.59	0.31-1.11	0.104
	30-35	1.58	1.08-2.31	**0.018**	0.81	0.28-2.30	0.685
	Above 35	1.40	0.86-2.27	0.174	0.14	0.03-0.70	**0.016**
**HbA1C ^2^**	Below 8	2.02	1.34-3.03	**0.001**	0.86	0.25-2.98	0.805
	Above 8	2.28	0.71-7.33	0.164	NA		

Boldface type indicates p<0.05.

Reference group: PO intake. Number of patients: PO = 2,358, IV/SC = 602.

HR, hazard ratio; CI, confidence interval; Pv = P-value.

^a^ Multivariable Cox regression, adjusted to left hip T score, patient sex, age at DM and osteoporosis diagnosis, fracture in femur.

^b^ Multivariable Cox regression, adjusted to left hip T score, patient sex, age at DM and osteoporosis diagnosis, and time from treatment initiation to fracture event.

Number of patients in each group: ^1^below 30 = 1,743, above 30 = 799, above 35 = 418; ^2^below 8 = 529, above 8 = 64.

BMI, body mass index; CI, confidence interval; HbA1c, glycated hemoglobin; HR, hazard ratio; IV/SC, intravenous or subcutaneous; PO, oral.

## 4 Discussion

In this large, real-world, propensity score–matched cohort of patients with diabetes and osteoporosis, we found that oral antiresorptive treatment was significantly associated with improved overall survival but not with a reduction in fracture risk. Despite the absence of a significant difference in major osteoporotic fracture incidence between treated and untreated groups, the 14% reduction in mortality observed among treated patients suggests benefits beyond fracture prevention. This survival advantage remained consistent across multiple clinical subgroups, including those aged >75 years, individuals with lower BMI, and those with impaired renal function. The association may reflect broader systemic effects of osteoporosis treatment, potentially mediated through anti-inflammatory mechanisms, reductions in frailty, or improvements in musculoskeletal integrity (21–23). Alternatively, it may serve as a proxy for greater engagement with healthcare services among treated patients. This interpretation aligns with previous studies demonstrating a mortality benefit associated with osteoporosis therapy in high-risk populations (24–26).

In contrast, the lack of significant differences in fracture outcomes may be explained by competing risks of death or residual confounding not fully addressed by the propensity score matching. Moreover, the analysis was based on treatment initiation rather than cumulative exposure, and oral treatment may not have been consistently taken, potentially diluting observed effects. It is well established that adherence and persistence with oral bisphosphonates are generally low in real-world settings (27–29). This may have contributed to the lack of observed fracture benefit in the orally treated group, as poor compliance could attenuate treatment effectiveness despite initiation.

When comparing route of administration, IV/SC therapies were associated with a significantly lower fracture risk compared to oral agents but paradoxically with higher all-cause mortality. This discrepancy may reflect confounding by indication, as clinicians may preferentially prescribe IV/SC therapies to patients with more advanced disease, poor gastrointestinal tolerance, or greater comorbidity burden—all of which are independently associated with higher mortality. Although our models adjusted for key variables including eGFR, T-score, and comorbidities, unmeasured factors such as frailty may still account for this difference. Importantly, in Israel, IV/SC therapies are also considered first-line treatment following hip fractures, further enriching this group with individuals at inherently higher risk. Therefore, the observed mortality difference likely reflects underlying baseline risk not fully captured by matching, rather than a harmful effect of the injectable therapies themselves. The enhanced fracture protection, especially among patients with BMI >35, supports the potency and adherence-independent advantage of parenteral agents (30).

This study has several notable strengths. It is a large real-world analysis which evaluates the effectiveness of osteoporosis treatment in patients with diabetes and obesity, a population often underrepresented in clinical trials. The use of a national registry allowed for a diverse, unselected cohort, enhancing generalizability. Moreover, propensity score matching and multivariable time-dependent Cox models were employed to reduce confounding and reflect treatment dynamics over time.

However, several limitations should be acknowledged. First, the analysis was based on treatment initiation rather than cumulative exposure, which may not reflect actual medication adherence. This is particularly relevant for oral bisphosphonates, which are known to have poor adherence and persistence in real-world settings, potentially attenuating observed fracture benefits. That lack of adherence and persistence data may lead to exposure misclassification, particularly underestimating the effectiveness of oral therapies. Still, in routine practice, adherence to oral osteoporosis therapies is known to be poor, whereas injectable therapies typically ensure better compliance. Therefore, the differences we observed between treatment groups may reflect real-world effectiveness, where patients prescribed oral and injectable agents ultimately experience different outcomes. Although we cannot determine whether these differences arise from lower adherence to oral agents, lower biological efficacy, or both, the findings may reflect the actual clinical scenario, which aligns with the aims of this real-world study.

Second, as an observational study, the potential for residual confounding remains, particularly with respect to frailty, functional status, or undiagnosed comorbidities that may influence both treatment decisions and outcomes. Third, fracture ascertainment was based on registry coding, which may underdetect clinically silent fractures. The lack of radiographic confirmation may result in under-ascertainment, particularly for asymptomatic or morphometric vertebral fractures. Although this may lower the overall sensitivity of fracture detection, it is unlikely to introduce differential bias across treatment groups.

Fourth, missing data for key covariates such as eGFR and BMD measurements may have introduced selection bias, although this was balanced across matched groups. Lastly, the observed increased mortality in IV/SC users is likely influenced by confounding by indication, as these therapies are often prescribed to more severely ill patients.

Overall, our results showed that antiresorptive treatment was significantly associated with improved overall survival, but only SC/IV treatment was associated with a reduction in fracture risk. These findings are relevant given the complex relationship between bone health and metabolic dysfunction in diabetes, as well as the underuse of osteoporosis treatments in this vulnerable group. They reinforce the importance of personalized treatment strategies for patients with diabetes and obesity and call for careful consideration of the optimal mode of drug administration in real-world clinical settings.

## 5 Conclusion

In this large, real-world cohort of patients with type 2 diabetes, oral antiresorptive therapy was associated with improved overall survival, though it did not significantly reduce fracture risk. In contrast, injectable therapies (IV/SC) offered greater protection against fractures compared to oral agents. The limited impact of oral therapy on fracture outcomes may be partially explained by poor adherence in routine clinical practice. These findings underscore the need for individualized osteoporosis management strategies in high-risk diabetic populations.

## Data Availability

The raw data supporting the conclusions of this article will be made available by the authors, without undue reservation.

## Supplementary material

Supplementary Figure 1 Covariate balance following propensity score matching (Treated vs. Not treated). The X axis represents the standardized mean difference (SMD) of each variable between patients receiving treatment to those who didn’t before (red) and after (blue) propensity score matching. SMD should be as close to 0 as possible, between -0.1 to 0.1 is considered good.

Supplementary Figure 2 Covariate balance following propensity score matching (PO vs. IV/SC). The X axis represents the standardized mean difference (SMD) of each variable between patients receiving treatment to those who didn’t before (red) and after (blue) propensity score matching. SMD should be as close to 0 as possible, between -0.1 to 0.1 is considered good.
