# Correction: Fracture and mortality outcomes by osteoporosis treatment route in patients with type 2 diabetes and obesity: a propensity-matched registry study

**DOI:** 10.3389/fendo.2026.1867225

**Published:** 2026-05-12

**Authors:** Vanessa Rouach, Ohad Regev, Hilary Gortler, Yona Greenman, Gabriel Chodick, Inbal Goldshtein

**Affiliations:** 1 Institute of Endocrinology, Diabetes, Hypertension and Metabolism, Sourasky Medical Center, Tel Aviv, Israel; 2 Epidemiology Department, School of Public Health, Tel Aviv University, Tel Aviv, Israel; 3 Joyce and Irving Goldman Medical School, Faculty of Health Sciences, Ben-Gurion University of the Negev, Beer Sheva, Israel; 4 Department of Public Health, Faculty of Health Sciences, Ben-Gurion University of the Negev, Beer Sheva, Israel; 5 Gray Faculty of Medical and Health Sciences, Tel Aviv University, Tel Aviv, Israel; 6 Maccabitech Institute for Research and Innovation, Maccabi Healthcare Services, Tel Aviv, Israel; 7 Dina Recanati School of Medicine, Reichman University, Herzliya, Israel

**Keywords:** anti-resorptive treatments, fracture risk, mortality, obesity, osteoporosis, type 2 diabetes mellitus

The reference for “1. Schwartz AV, Sellmeyer DE, Ensrud KE, Cauley JA, Tabor HK, Schreiner PJ, et al. Older women with diabetes have an increased risk of fracture: a prospective study. J Clin Endocrinol Metab. 2001;86(1):32–38. doi:10.1210/jcem.86.1.7139.” was erroneously written as “1. Schwartz AV, Sellmeyer DE, Ensrud KE, Cauley JA, Tabor HK, Schreiner PJ, et al. Older women with diabetes have an increased risk of fracture: a prospective study. J Clin Endocrinol Metab. (2001) 86:32–8. doi:10.1210/jcem.86.1.7098.”

The reference for “4. Premaor MO, Pilbrow L, Tonkin C, Parker RA, Compston J. Obesity and fractures in postmenopausal women. J Bone Miner Res. 2010;25(2):292–297. doi:10.1359/jbmr.091004.” was erroneously written as “4. Premaor MO, Pilbrow L, Tonkin C, Parker RA, Compston J. Obesity and fractures in postmenopausal women. J Bone Miner Res. (2010) 25:292–7. doi:10.1359/jbmr.090807.”

The reference for “9. McClung MR, Lewiecki EM, Cohen SB, Bolognese MA, Woodson GC, Moffett AH, et al. Denosumab in postmenopausal women with low bone mineral density. N Engl J Med. 2006;354(8):821–831. doi:10.1056/NEJMoa044459.” was erroneously written as “9. McClung MR, Lewiecki EM, Cohen SB, Bolognese MA, Woodson GC, Moffett AH, et al. Denosumab in postmenopausal women with low bone mineral density. J Clin Endocrinol Metab. (2006) 91:3941–7. doi: 10.1210/jc.2006-0610.”

The reference for “10. Viggers R, Al-Mashhadi Z, Starup-Linde J, Vestergaard P. The efficacy of alendronate versus denosumab on major osteoporotic fracture risk in elderly patients with diabetes mellitus: a Danish retrospective cohort study. Front Endocrinol (Lausanne). 2021;12:826997. doi:10.3389/fendo.2021.826997.” was erroneously written as “10. Zizolfi B, Ciaponi F, Gonnelli S, Caffarelli C, Merlotti D, Nuti R, et al. Alendronate vs denosumab: similar fracture outcomes in subjects with diabetes. Front Endocrinol. (2021) 12:826997. doi:10.3389/fendo.2021.826997.”

The reference for “11. Viggers R, Starup-Linde J, Vestergaard P. Discrepancies in type of first major osteoporotic fracture and anti-osteoporotic therapy in elderly people with type 2 diabetes mellitus: a retrospective Danish cohort study. Bone. 2023;171:116745. doi:10.1016/j.bone.2023.116745.” was erroneously written as “11. Viggers R, Al Mashhadi Z, Starup Linde J, Vestergaard P. The efficacy of alendronate versus denosumab on major osteoporotic fracture risk in elderly patients with diabetes mellitus. Bone. (2022) 171:116745. doi:10.1016/j.bone.2022.116745.”

The reference for “12. Eastell R, Vittinghoff E, Lui LY, Ewing SK, Schwartz AV, Bauer DC, Black DM, Bouxsein ML, et al. Diabetes mellitus and the benefit of antiresorptive therapy on fracture risk. J Bone Miner Res. 2022;37(11):2121–2131. doi:10.1002/jbmr.4697.” was erroneously written as “12. Lane NE, Black DM, Cummings SR, Eastell R, Schwartz AV, Bauer DC, et al. Pooled analyses of bisphosphonate trials show equivalent fracture risk reduction. J Bone Miner Res. (2022) 37:2121–34. doi:10.1002/jbmr.4686.”

The reference for “28. Ziller V, Kostev K, Kyvernitakis I, Boeckhoff J, Hadji P. Persistence and compliance of medications used in the treatment of osteoporosis—analysis using a large-scale, representative, longitudinal German database. Int J Clin Pharmacol Ther. 2012;50(5):315–322. doi:10.5414/CP201632.” was erroneously written as “28. Hadji P, Ziller V, Gothe H, Becker C, Linder R, Kostev K. Persistence with oral and parenteral osteoporosis therapies: analysis of claims data. Osteoporos Int. (2013) 24:917–23. doi:10.1007/s00198-012-2020-x.”

The reference for “29. Rabenda V, Mertens R, Fabri V, Vanoverloop J, Sumkay F, Vannecke C, et al. Adherence to bisphosphonate therapy and hip fracture risk in osteoporotic women. Osteoporos Int. 2008;19:811–818. doi:10.1007/s00198-007-0506-x.” was erroneously written as “29. Rabenda V, Mertens R, Fabri V, Vanoverloop J, Sumkay F, Vannecke C, et al. Adherence to bisphosphonates therapy and fracture risk. J Bone Miner Res. (2008) 23:1419–25. doi:10.1359/jbmr.080516.”

The reference for “30. Tsai YL, Wu CH, Li CC, Shih CA, Chang YF, Hwang JS, Tai TW. Drug adherence and treatment duration for denosumab and mortality risk among hip fracture patients. Osteoporos Int. 2023;34(10):1783–1791. doi:10.1007/s00198-023-06845-0.” was erroneously written as “30. Hsueh YH, Su CM, Lai CH, Lin CL, Lin WL. Association between denosumab adherence and mortality in patients with hip fractures. J Clin Endocrinol Metab. (2023) 108:2793–801. doi:10.1210/clinem/dgad475.“

The original version of this article has been updated.

