# Supplementary figures and images for: Fracture and mortality outcomes by osteoporosis treatment route in patients with type 2 diabetes and obesity: a propensity-matched registry study
Source: Front Endocrinol (Lausanne). 2026 Feb 3;17:1688669. doi: 10.3389/fendo.2026.1688669 (PMC12909188; doi:10.3389/fendo.2026.1688669)

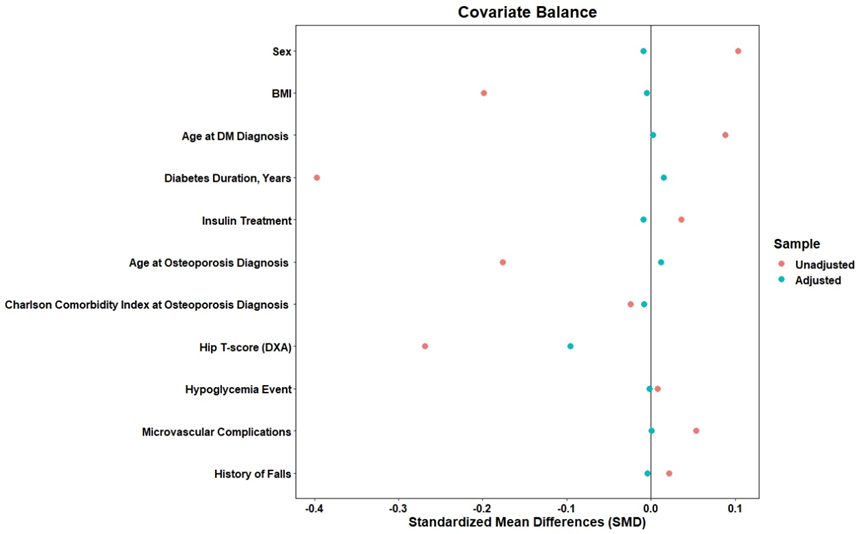

Supplement: Supplementary Figure 1 — Covariate balance following propensity score matching (Treated vs. Not treated). The X axis represents the standardized mean difference (SMD) of each variable between patients receiving treatment to those who didn’t before (red) and after (blue) propensity score matching. SMD should be as close to 0 as possible, between -0.1 to 0.1 is considered good. [file Image1.png]

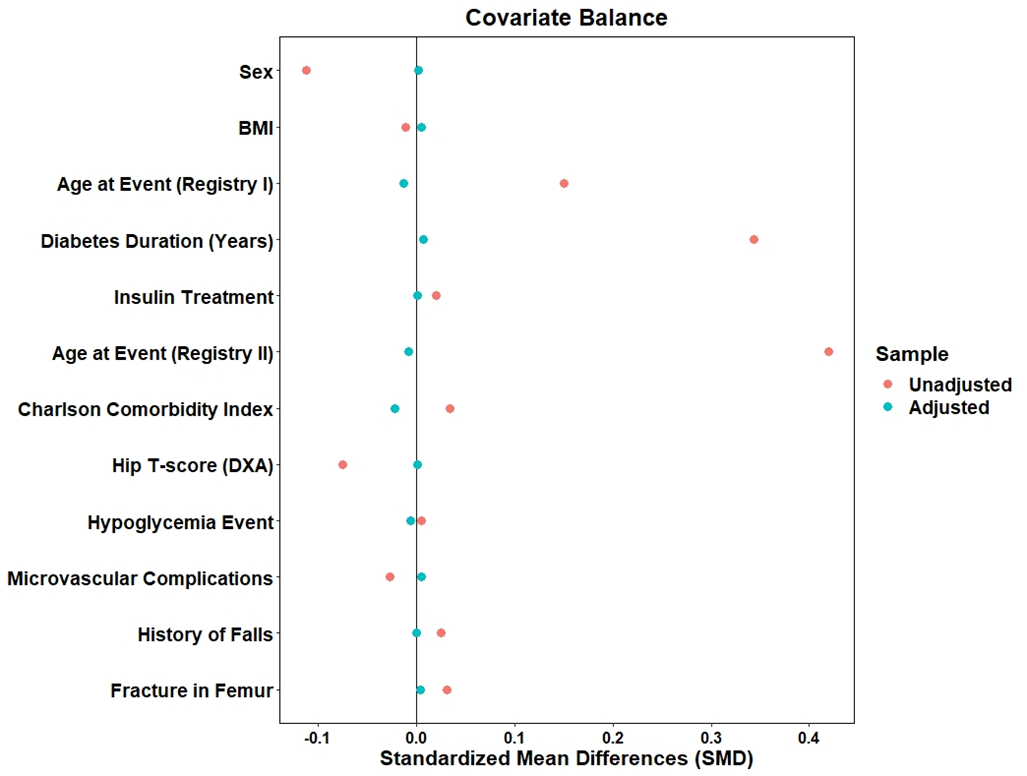

Supplement: Supplementary Figure 2 — Covariate balance following propensity score matching (PO vs. IV/SC). The X axis represents the standardized mean difference (SMD) of each variable between patients receiving treatment to those who didn’t before (red) and after (blue) propensity score matching. SMD should be as close to 0 as possible, between -0.1 to 0.1 is considered good. [file Image2.png]
